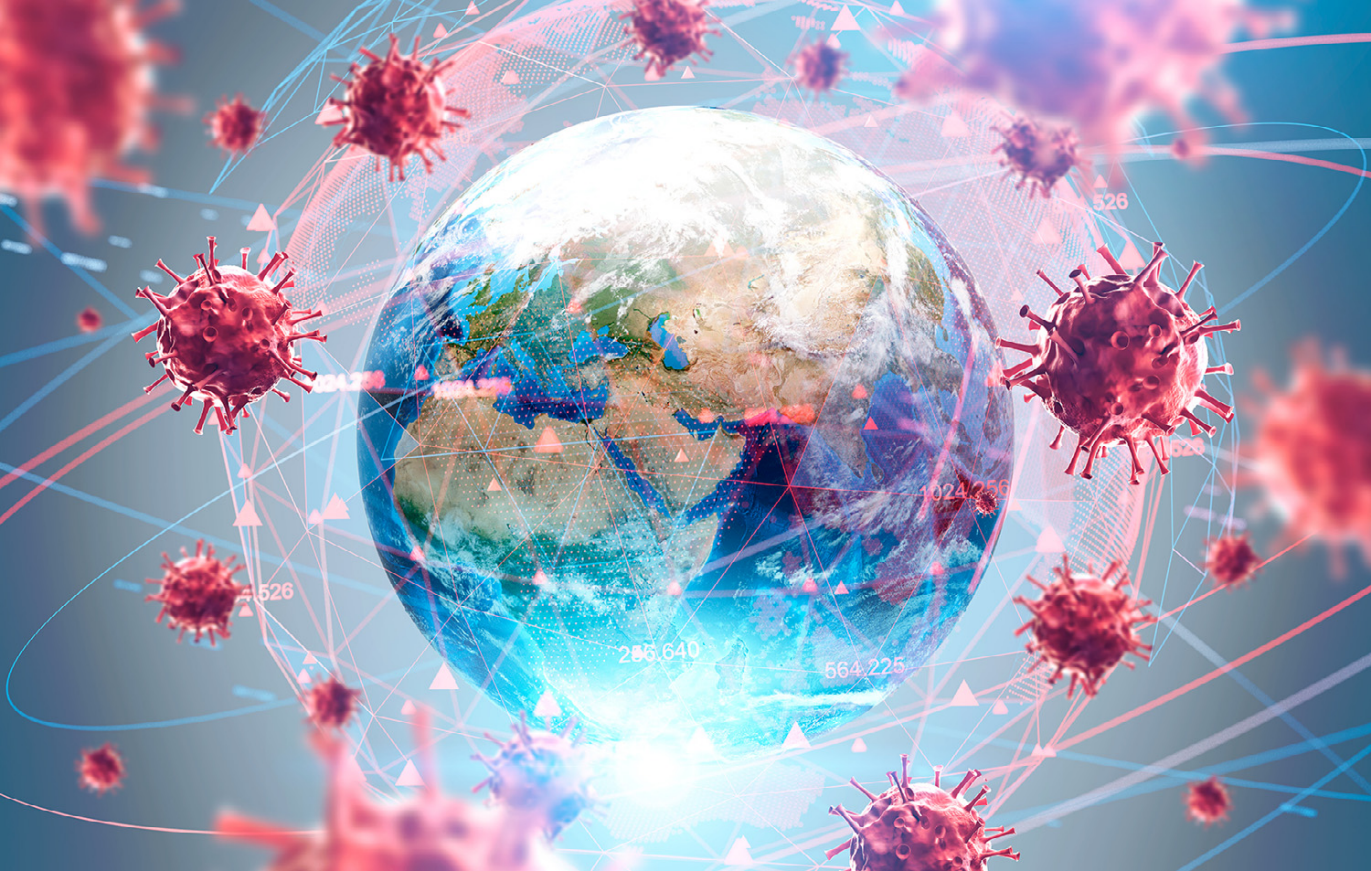# The future belongs to the globalists

**DOI:** 10.1016/j.eclinm.2020.100406

**Published:** 2020-05-29

In a matter of 6 months the COVID-19 pandemic overtook the world as we knew it, affected our normal social, economic, and health-care systems and changed the way we viewed infectious disease and spread. The estimated global mortality from COVID-19 has passed the 300 000 mark and, in an effort to save lives, the resultant lockdowns and travel bans have caused economies to suffer their most severe contractions in the first quarter of the year in more than a decade. As governments look to lifting restrictions in hopes of rebolstering the economy and returning societies to normal, we ask, should we be striving for normality as we knew it?

On the rise of right-wing nationalistic politics, where many countries looked to isolate themselves, such as US President Donald Trump's America first campaign or Brexit, the COVID-19 pandemic has shown how the world is indisputably connected. The extent of connectedness was portrayed in the unprecedented speed at which the virus spread. China notified WHO about a cluster of pneumonia-like cases on Dec 31, 2019, and on Jan 13, 2020, the first confirmed case outside of China was declared in Thailand, and the first European case in France on Jan 24, 2020. With reported cases in at least 187 countries, many of which do not have the health-care facilities or government infrastructure to handle COVID-19, the need for a more global outlook and strategy is becoming apparent. China now reports more imported cases of COVID-19 than locally transmitted cases, and as travel bans begin to ease, fears of a second wave of infection are appearing, unless all countries can contain the virus.

Collaboration and solidarity between countries is not always guaranteed. As Italy became the new epicentre of the disease, the EU failed to respond to the country's request for aid via the bloc's emergency mechanism, as countries imposed their own restrictions, banned the export of medical supplies, and were uncoordinated in their approach. On April 16, 2020, Ursula von der Leyen, president of the European Commission, addressed the EU parliament and issued a “heartfelt apology” on behalf of Europe for their “only-for-me” response, and admitted they did not stand by Italy when they needed it. The European Commission laid out their new plans, including locating up to €100 billion to the hardest-hit countries, starting with Italy and loans guaranteed by all member states to show Europe's solidarity. The UK Government has also been accused of prioritising country politics over intercountry cooperation, because they failed to join the EU emergency scheme, initiated by the European Commission, which uses the bulk-buying power of the 500 million-person single market to get priority for ventilators and protective equipment. Mounting criticism led to a reversal in approach, with a spokesperson reporting that the government will now consider participating in future procurement schemes. These delays reflect the sometimes reluctant departure from prepandemic ideologies in favour of a more unified approach—a majority of the time because of increasing criticism and a demand for better management.

The COVID-19 pandemic has not only highlighted the insufficient response of international aid, but also the inabilities of individual countries to deal with a health crisis. Despite COVID-19 initially hitting developed and wealthy nations, health-care systems struggled to cope with the increase in demand, with only a few countries able to contain the virus and flatten their curve before catastrophic death tolls. In many cases, this success is strongly correlated to the strength of a country's health-care system, as seen in the 2019 Legatum Prosperity Index which measures economic and social prosperity policies and conditions based on 12 pillars in 167 nations. Among those ranked in the top ten in the health pillar—a specific measurement looking at the extent to which people in each country are healthy and have access to services—are Singapore (1^st^), Japan (2^nd^), South Korea (4^th^), and Hong Kong (6^th^). With a population of 41 million, South Korea has 11 065 cases and 263 deaths attributed to COVID-19. Alongside mass testing and contact tracing, the South Korean health-care system was particularly prepared after being criticised for their management of the Middle-East respiratory syndrome (MERS) in 2015 which led to the largest outbreak outside the Middle East. The system is funded by a compulsory National Health Insurance Scheme that covers 97% of the population. Germany, which has managed to maintain a lower COVID-19 mortality rate than most of its neighbouring countries, ranked 12th and in the past has been criticised for having too many hospitals. Before the crisis Germany had 33 · 9 intensive care beds per 100 000 people, compared with 9 · 7 in Spain and 8 · 6 in Italy. Universal health coverage of some kind seems imperative in managing and treating the majority of the population, and countries that have done little in the past to strengthen their health system, such as the USA, have been those most affected by the SARS-CoV-2 virus. COVID-19 has highlighted what is truly necessary and important to be an advanced and successful country, because, ultimately, the health and welfare of all members of society should be valued, funded, and appreciated.

COVID-19 has disrupted the world and has challenged the infrastructure of global society. The previous economic and political constructs that seemed immovable and constant have fallen, but with that, an opportunity to re-evaluate what we want to prioritise as a civilisation has risen. Health care as a basic human right has never been more apparent, as has the lack of funding of the health-care sector in many countries. Financial instability and fear have historically contributed to feelings of xenophobia and hate, and with COVID-19 we have seen the rise of hate crimes against minority groups and racist vitriol. In September 2019, US President Donald Trump claimed to the UN General Assembly that “the future doesn't belong to globalists. The future belongs to patriots.” However, now more than ever, the need to reject these ideologies is imperative when our personal safety is reliant on the health and safety of others, and the marginalisation of any sector of society will only lead to more chaos and disruption. Life as we knew it has shown to be unsustainable, unsafe, and unjustifiable, and now our survival depends on our ability to learn and evolve.

*EClinicalMedicine*

**Figure:**